# Optimizing the Interaction of Exercise Volume and Metformin to Induce a Clinically Significant Reduction in Metabolic Syndrome Severity: A Randomised Trial

**DOI:** 10.3390/ijerph17103695

**Published:** 2020-05-24

**Authors:** Joyce S. Ramos, Lance C. Dalleck, Caitlin E. Keith, Mackenzie Fennell, Zoe Lee, Claire Drummond, Shelley E. Keating, Robert G. Fassett, Jeff S. Coombes

**Affiliations:** 1 Caring Futures Institute & SHAPE Research Centre, Exercise Science and Clinical Exercise Physiology, College of Nursing and Health Sciences, Flinders University, Room S268, South Wing, Sturt Building, Bedford Park, Adelaide 5042, South Australia, Australia; ldalleck@western.edu (L.C.D.); caitlin.keith@flinders.edu.au (C.E.K.); mackenzie.fennell@flinders.edu.au (M.F.); zoe.lee@flinders.edu.au (Z.L.); claire.drummond@flinders.edu.au (C.D.); 2 Centre for Research on Exercise, Physical Activity and Health, School of Human Movement and Nutrition Sciences, The University of Queensland, Brisbane 4072, Queensland, Australia; s.keating@uq.edu.au (S.E.K.); r.fassett@uq.edu.au (R.G.F.); jcoombes@uq.edu.au (J.S.C.); 3 Recreation, Exercise, and Sport Science Department, Western Colorado University, Gunnison, CO 81231, USA

**Keywords:** metabolic syndrome, exercise volume, metformin, interval training

## Abstract

Insulin resistance is a central mediating factor of the metabolic syndrome (MetS), with exercise training and metformin proven antidotes to insulin resistance. However, when the two therapies are combined there is conflicting data regarding whether metformin blunts or improves exercise training-induced adaptations. The volume of exercise (duration, intensity, and frequency) on the interaction of exercise training and metformin has yet to be investigated. The aim of this study is therefore to explore the impact of a combination of different exercise volumes and metformin on MetS severity. This is a secondary analysis of data from one of the sites of the ‘Exercise in Prevention of Metabolic Syndrome’ (EX-MET) study. Ninety-nine adults with MetS were randomized into a 16-week exercise program completing either: (i) moderate-intensity continuous training (MICT) at 60–70% of peak heart rate (HRpeak) for 30 min/session (*n* = 34, 150 min/week); (ii) high-volume high-intensity interval training (HIIT) consisting of 4 × 4 min bouts at 85–95% HRpeak, interspersed with 3 min of active recovery at 50–70% HRpeak (*n* = 34, 38 min/session, 114 min/week); or (iii) low volume HIIT, 1 × 4 min bout of HIIT at 85–95% HRpeak (*n* = 31, 17 min/session, 51 min/week). Metformin intake was monitored and recorded throughout the trial. MetS severity was calculated as z-scores derived from MetS risk factors assessed at pre- and post-intervention. Sixty-five participants had complete pre- and post-intervention data for MetS z-score, of which 18 participants (28%) were taking metformin. Over the 16-week intervention, a similar proportion of participants clinically improved MetS severity (Δ ≥ −0.87) with metformin (8/18, 44%) or without metformin (23/47, 49%) (*p* = 0.75). While there were no between-group differences (*p* = 0.24), in those who did not take metformin low-volume HIIT had more likely responders (10/15, 67%) compared to MICT (6/16, 38%) and high-volume HIIT (7/16, 44%). In those taking metformin, there was a lower proportion of participants who clinically improved MetS severity following high-volume HIIT (1/6, 17%) compared to MICT (2/4, 50%) and low-volume HIIT (5/8, 63%), but with no between-group difference (*p* = 0.23). Moreover, in those who performed high-volume HIIT, there was a statistically significantly higher proportion (*p* = 0.03) of likely non-responders with improved MetS severity in participants taking metformin (4/6, 67%) compared to those not taking metformin (3/16, 19%). In individuals with MetS, the effect of high volume HIIT on MetS severity may be blunted in those taking metformin. These findings need to be confirmed in a larger study.

## 1. Introduction

Metabolic syndrome (MetS) is the cluster of cardiovascular disease (CVD) risk factors including central obesity, impaired glucose metabolism (prediabetes), dyslipidemia, and hypertension, significantly predisposing an individual to type 2 diabetes mellitus (T2D) and subsequent CVD-related morbidity and mortality [1]. Insulin resistance is also purported to be the central mediating factor of MetS [2], making it a viable target for the amelioration of MetS severity, and thus risk of T2D and CVD.

Lifestyle intervention including habitual exercise is considered the first-line therapy for improving insulin resistance or glycemic control and overall cardiometabolic health in those at risk of T2D (prediabetes) and CVD [3]. Specifically, exercise has been shown to have beneficial effects on cardiovascular risk factors constituting the MetS including central obesity, hypertension, hypertriglyceridemia, low HDL-cholesterol, and hyperglycemia [4]. However, long-term adherence to exercise remains problematic [5,6], resulting in increased reliance on pharmacological therapy to maintain normoglycemia. It is therefore currently recommended by the American Diabetes Association (ADA) [7] and the Canadian Diabetes Association [8] that, in addition to lifestyle modification, those with impaired glucose metabolism or insulin resistance should be considered for metformin, an anti-hyperglycaemic pharmacological medication. The underlying premise of this recommendation is that combined lifestyle and pharmacological treatment will be more effective than either intervention alone. However, despite the frequency of co-prescription, the potential influence of their interaction on improvements in health is relatively unclear [9].

Given the link between insulin resistance and CVD risk factors, metformin is not only used to treat or manage impaired glucose tolerance but is also purported to lower CVD risk [10]. An exercise-induced increase in cardiorespiratory fitness (CRF) has also long been established as a cost-effective antidote, with fitter individuals with MetS having less risk of cardiovascular mortality relative to lower fit counterparts [11]. However, the combined effects of exercise training and metformin on CVD risk are equivocal [12], perhaps due to inconsistent outcome measures used between studies. Recently, a continuous measure of MetS severity (MetS z-score) has been shown to be a useful and sensitive biomarker to help detect responses to treatments. It is a continuous risk score assessment scale to give credit for improvement in a specific MetS feature, if improvement to move out of a “qualifying” category is not achieved [13]. For example, a decrease in fasting blood glucose from 6.1 mmol/L to 5.8 mmol/L following an intervention would still be classified as a MetS risk factor according to the International Diabetes Federation (IDF) criteria (fasting glucose ≥ 5.6 mmol/L) [1]. It has been reported that a one-year change in MetS z-score is strongly associated with risk of developing T2D and CVD within 1–5 years [14]. A one-year lifestyle intervention including brisk walking for 150 min per week induced a greater reduction in MetS z-score compared to metformin (850 mg, twice daily) [14]. However, the combined effect of exercise and metformin on MetS z-score remains unclear.

Malin et al. [15] was the first group to compare the efficacy of a 12-week combination of exercise training (~190 min/week, 3 days/week, 60–75 min per session, 70% peak heart rate (HRpeak) and metformin (2000 mg/day), relative to exercise or metformin only on MetS severity (ATP-III score). They found that metformin appeared to blunt the effect of the exercise training. Given that higher volume exercise (23 kcal/kg/week) has been demonstrated to induce a greater reduction in MetS z-score relative to lower volume exercise (14 kcal/kg/week) [16], it is important to determine the interaction between metformin and higher exercise volumes. Conversely, in those unable to perform higher volume exercise, it would be important to determine the minimum exercise volume that can be prescribed in combination with metformin to induce a clinically meaningful MetS severity reduction. The aim of this study was, therefore, to explore the impact of a combination of different exercise intensities and volumes and metformin on MetS severity.

## 2. Materials and Methods

### 2.1. Participants and Study Design

**Figure 1:**
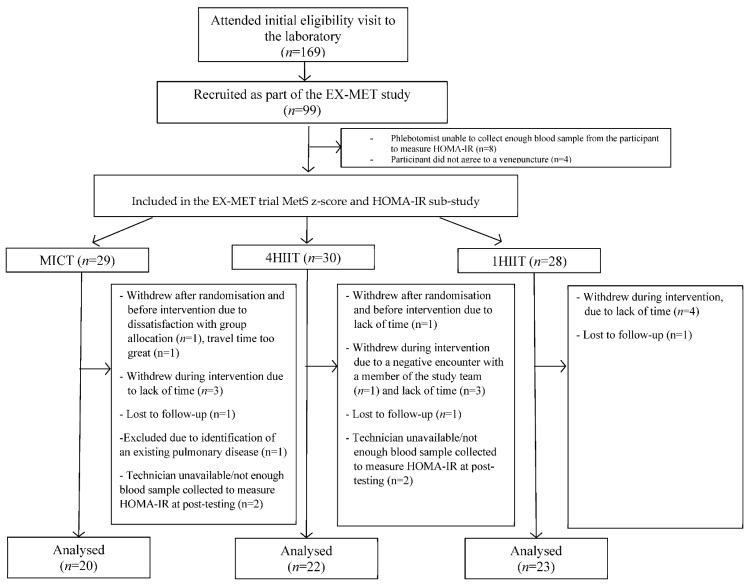
Participant flow chart. MICT, moderate-intensity continuous training; 4HIIT, 4 × 4 min high-intensity interval training; 1 × 4 min high-intensity interval training.

Ninety-nine people with MetS diagnosed according to the International Diabetes (IDF) criteria were recruited between January 2013 and August 2015. The present study is a sub-study of the Exercise in Prevention of Metabolic Syndrome (EX-MET) multi-centre trial (ClinicalTrials.gov NCT01676870), which reports data collected exclusively at the Brisbane site. Included participants were randomized (stratified by age and sex) into one of three 16-week exercise programs (MICT, *n* = 34; 4HIIT, *n* = 34; 1HIIT, *n* = 31). Figure 1 presents a diagram of participant flow. Details on inclusion criteria and randomisation procedure have been reported previously [17]. The Medical Research Ethics committee of The University of Queensland (Brisbane, Australia) approved this study (ethics approval number 2012000617). Metformin medication dosage was recorded at baseline and post-intervention. Participants were also instructed to notify the research team of any dosage change throughout the intervention.

### 2.2. Metabolic Syndrome Severity and Insulin Resistance

The following tests were conducted in a fasted state (12 h) to determine the participants’ eligibility for the study: (i) fasting glucose (FG) level and lipid profile; (ii) resting brachial systolic blood pressure (SBP) and diastolic blood pressure (DBP); (iii) anthropometric measures (waist circumference and body mass index). Sex-specific MetS z-score was calculated using the following equations to determine MetS severity [18]:

MetS z-score_men_ = [(40 − HDL-C)/8. 9] + [(TG − 150/69)] + [(FG − 100)/17.8] + [(WC − 102)/11.5] + [(MAP − 100)/10.1].

MetS z-score_women_ = [(50 − HDL-C)/14.5] + [(TG − 150/69)] + [(FG − 100)/17.8] + [(WC − 88)/12.5] + [(MAP − 100)/10.1].

Insulin resistance was determined via the homeostasis model assessment of insulin resistance (HOMA-IR). This was calculated through the HOMA2 calculator version 2.2 [19].

### 2.3. Cardiorespiratory Fitness (CRF)

CRF was assessed during a graded maximal exercise test (GXT), determined as the peak oxygen uptake (VO_2_peak) measured via indirect calorimetry using a MetaMax II (Cortex, Leipzig, Germany) or Parvo Medics TrueOne 2400 system (Parvomedics Inc., Sandy, UT, USA). VO_2_peak was identified as the highest 15-s time averaged oxygen uptake. The GXTs were conducted either on a cycle or treadmill ergometer, depending on the participant’s preference during the supervised sessions, or on orthopaedic limitations. A nutritional supplement (Sustagen, 250 mL; Dutch Chocolate, Nestle, Gympie, Queensland, Australia) was required to be consumed by participants 2 h before the GXT in order to standardize nutrition. An 8-min warm-up consisting of 4-min stages (stage 1 warm-up: 4 km/h at 0% incline or 50–60 rpm at 0 W; stage 2 warm-up: 4 km/h at 4% incline or 50–60 rpm at 25 W) preceded all GXT to familiarize participants with the selected ergometer and protocol of the test. Following the warm-up period, the workload was slightly increased to begin the test (treadmill speed individualized at 4–6 km/h or cycling at 60–70 rpm, intensity; ~6 incline or 50 W). The load (2% incline or 25 W) and speed (individualized: 6–9 km/h or 60–70 rpm) increased every minute thereafter until exhaustion. A 12-lead electrocardiogram was used to monitor the participant throughout the GXT, with data recorded at rest and every 3 min. A medical practitioner supervised all GXTs at baseline. A tester blinded to the group allocation provided standardized verbal cues to motivate participants to achieve a maximal effort. The test was terminated if any contraindications developed [20].

### 2.4. Training Protocol

**Figure 2:**
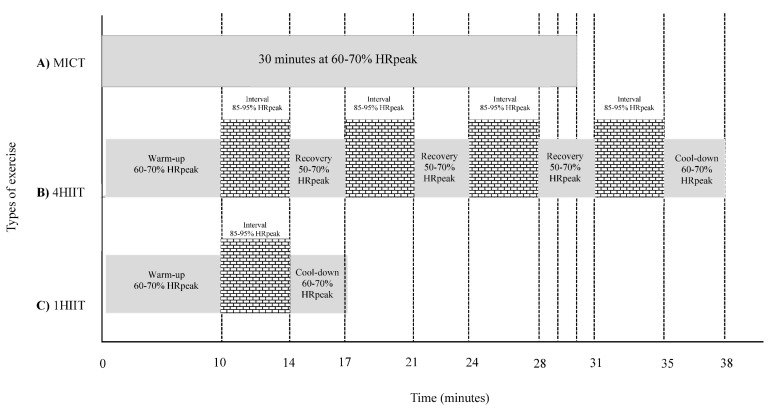
Schematic representation of protocols for moderate-intensity continuous training (MICT) (**A**), 4HIIT (**B**) and 1HIIT (**C**).

The HIIT and MICT groups trained three and five times per week, respectively (with at least a day between HIIT sessions). Participants were required to visit two supervised sessions with an exercise physiologist at the University of Queensland exercise laboratory. The remaining sessions were conducted in an unsupervised environment. Heart rate (HR) and rate of perceived exertion (RPE) were monitored throughout all sessions via a HR monitor (Polar Electro, Kempele, Finland) and the Borg 6–20 scale [21]. A training log was provided to participants to record HR and RPE during the unsupervised sessions. The supervised sessions were conducted either on a treadmill or cycle ergometer, as indicated by the participant’s preference or by orthopaedic limitations. The unsupervised sessions included outdoor/indoor pursuits that involved large muscle groups such as walking, running, rowing, and swimming. The MICT group trained continuously for 30 min per session at 60–70% HRpeak or RPE of 11–13 on the Borg scale, and with no signs of shortness of breath (Figure 2A). The 4HIIT and 1HIIT sessions started with a 10-min warm-up and finished with a 3-min cool down, with a total exercise duration of 38 and 17 min/session, respectively (Figure 2B,C). The 4HIIT protocol included four bouts of 4-min intervals at 85–95% HRpeak or RPE of 15–17 on Borg scale, separated by 3-min of active recover at 50–70% HRpeak (Figure 2B). The 1HIIT protocol consisted of one 4-min interval at 85–95% HRpeak or RPE of 15–17 on Borg scale (Figure 2C). All participants were instructed to achieve the target RPE or HR for each interval within the initial 2 min of the 4-min interval.

### 2.5. Statistical Analysis

Data were analysed using the SPSS version 25 software package (IBM, New York, NY, USA). Baseline values and training adherence between groups were compared using the One-way ANOVA or Kruskal-Wallis and Chi-square tests. Between-group difference in the change in continuous variables from pre- to post-intervention was analysed via analysis of covariance (ANCOVA), with the difference/Δ-value assigned as the dependent variables and the baseline value as the covariate. Continuous and categorical variables are reported as mean ± standard deviation (SD)/median (range) and frequencies, respectively.

Delta values (Δ) (post-intervention minus pre-intervention value) were calculated to determine individual MetS z-score training responsiveness. In order for a participant to be considered a likely responder to improvements in MetS z-score, they would need to have a MetS z-score change greater than the established categories in a favorable direction. To increase the confidence in response classification, a combination of technical error of measurement (TEM) and meaningful clinically important difference (MCID), where applicable, was used to determine the thresholds for response categories [22]. The categories included were likely responder (Not taking Metformin: >−0.87; Taking Metformin: >−1.3), likely non-responder (Not taking Metformin: >−0.33; Taking Metformin: >0.1), and uncertain (Not taking Metformin: −0.87 to −0.33; Taking Metformin: −1.3 to 0.1), to provide information on the variation of participant responses relative to the MCID. The TEMs for each training group either taking metformin or not taking metformin were estimated by multiplying the mean baseline value of MetS z-score [23] from a previously published co-efficient of variation. The MCID in MetS z-score was determined from previous studies as ≥−0.60 [14].

Chi-square tests were used to analyse the proportion of response classification for MetS z-score following the study period with a subsequent Cramer’s V test to quantify effect size. Significance level was set at *p* < 0.05.

## 3. Results

Out of the 99 participants recruited as part of the EX-MET trial from January 2013 to August 2015, 65 had complete pre- and post-intervention data for MetS z-score and HOMA-IR (Figure 1). Out of these 65 participants, 18 were prescribed metformin by their general practitioner. There was no change in their average metformin dose throughout the study (Pre vs. Post: 1000 mg (range 500 to 2000 mg) vs. 1000 mg (500 to 2000 mg) *p* = 0.32), with one participant changing dose during the study (Pre vs. Post: 1000 to 2000 mg). The physical and physiological characteristics at baseline and 16 weeks for participants who completed the present study are presented in Table 1, Table 2 and Table 3. At baseline, there was a significant difference (*p* < 0.05) in MetS z-score, glucose levels, and proportion of T2D between participants taking or not taking metformin (Table 1). Table 2 and Table 3 show no significant physical or physiological differences at baseline between exercise groups in those taking/not taking metformin. Participants in the MICT, high-volume HIIT, and low-volume HIIT groups completed 87 ± 14%, 88 ± 9%, and 88 ± 16% of the prescribed sessions (between groups, *p* = 0.75), with an estimated energy expenditure (exercise volume; kcal) of 14,149 ± 4113 kcal, 16,919 ± 7394 kcal, and 6274 ± 2544 kcal, respectively, over the 16-week program (group difference, *p* < 0.001). This equated to 884 ± 257 kcal/week, 1057 ± 462 kcal/week, and 392 ± 159 kcal/week, respectively (group difference, *p* < 0.001). There were no reported adverse events that were attributed to the prescribed exercise programs in participants included in this sub-study. Table 1, Table 2 and Table 3 present no statistically significant changes in physical and physiological variables from pre- to post-intervention between groups, except for Table 1 showing a greater reduction in SBP following exercise training for those not taking metformin compared to participants taking metformin.

### 3.1. Inter-Individual Variability in MetS Severity Change

**Figure 3:**
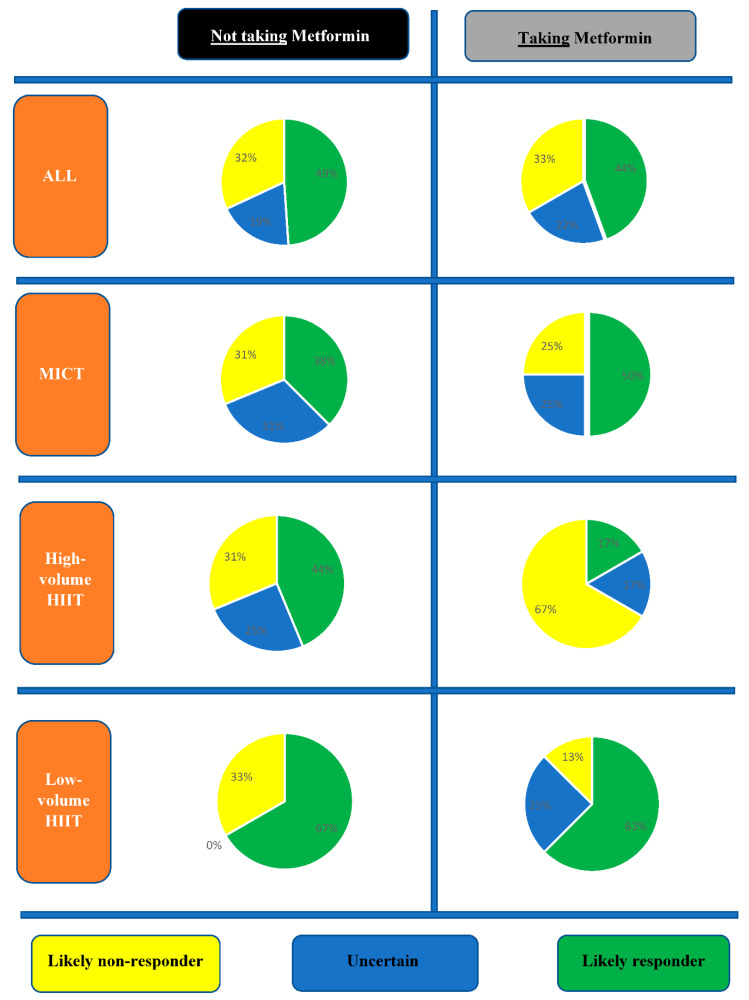
Proportions of response categories in metabolic syndrome (MetS) z-score change following exercise interventions in participants not taking or taking metformin.

Figure 3 presents the proportions of likely responders, likely non-responders, and uncertain, to a change in MetS z-score. When comparing participants not taking metformin vs. those taking metformin there were similar proportions of likely responders (23/47, 49% vs. 8/18, 44%; *p* = 0.75), likely non-responders (15/47, 32% vs. 6/18, 33%; *p* = 0.91, and uncertain (9/47, 19% vs. 4/18, 22%; *p* = 0.40). When comparing exercise volumes in those not taking metformin, although there were no statistically significant between-group (exercise volumes) differences (*p* = 0.24), low-volume HIIT had more likely responders (10/15, 67%) compared to MICT (6/16, 38%) and high-volume HIIT (7/16, 44%) (Figure 3). In those taking metformin, high-volume HIIT had less likely responders (1/6, 17%) relative to MICT (2/4, 50%) and low-volume HIIT (5/8, 63%), but with no statistically significant difference between exercise volume groups (*p* = 0.23). Moreover, in those who performed high-volume HIIT, there was a significantly higher proportion (*p* = 0.03) of likely non-responders in participants taking metformin (4/6, 67%) relative to those not taking metformin (3/16, 19%).

### 3.2. Inter-Individual Variability in Cardiorespiratory Fitness Changes

There were comparable proportions of participants classified as likely responders (8/44, 18% vs. 3/18, 17%; *p* = 0.89), likely non-responders (23/44, 52% vs. 8/18, 44%; *p* = 0.58), and uncertain (12/44, 27% vs. 7/18, 39%; *p* = 0.37) between those taking metformin or not taking metformin. In those not taking metformin, there was a greater proportion of likely responders to CRF improvement following high-volume HIIT (4/14, 29%) compared to MICT (2/16, 13%) and low-volume HIIT (2/14, 14%), but with no statistical significance between-group (exercise volume) difference (*p* = 0.47). In those taking metformin, there were more likely non-responders following MICT (3/4, 75%) relative to high-volume HIIT (2/6, 33%) and low-volume HIIT (3/8, 38%) (between exercise volume group difference, *p* = 0.37), with only the high-volume HIIT reporting likely responders to an increase in CRF (3/6, 50%) (between-group difference, *p* = 0.03).

## 4. Discussion

This is the first study to investigate the inter-individual changes in MetS severity following different exercise volumes in individuals with MetS who are either not taking or taking metformin medication. In participants taking metformin and who completed high-volume HIIT, there was a significantly higher proportion of likely non-responders to a clinically significant MetS severity reduction (4/6, 67%) compared to participants who were not taking metformin (3/16, 19%). This finding needs to be tested in a larger group.

A potential mechanism that explains the antagonising effect of metformin on the high-volume HIIT-induced improvement in MetS z-score found in the present study could be its capacity to inhibit skeletal muscle mitochondrial biogenesis and respiration [24]. Konopka et al. [24] showed that metformin abolished the exercise-mediated increase in skeletal muscle mitochondrial respiration, which was associated with an attenuated increase in insulin sensitivity and VO_2_max following the 12-week aerobic exercise program (45 min per session (15 min at 60% HRmax; 30 min 65–85%), 3x per week). Both high-volume [25,26] and high-intensity exercise [27,28] as reflected by the high-volume HIIT protocol in the present study increases mitochondrial biogenesis and this peripheral adaptation is associated with benefits to components of MetS such as IR and lower blood pressure. However, metformin inhibits mitochondrial respiration at complex I of the electron transport system [29,30] which consequently suppresses the key enzyme connecting oxidative phosphorylation and glycolysis (mitochondrial glycerophosphate dehydrogenase), ultimately leading to raised lactate due to the inhibition of gluconeogenesis [31,32]. In addition to this metformin-induced raised plasma lactate, metformin has also been demonstrated to increase HR and perceived exertion during exercise [33,34], which may all collectively result in the underestimation of the prescribed target HR and RPE in order to reach the desired metabolic challenge required to induce favorable metabolic adaptations.

When combining all exercise volumes, we found a similar proportion of participants categorised as likely responders to a clinical meaningful change in MetS z-score not taking (23/47, 49%) or taking metformin (8/18, 44%). This finding is consistent with a previous study [15] which also found no additive effect of metformin on exercise training-induced reductions in MetS severity. However, it is in contrast with studies suggesting that combined lifestyle modification (including exercise, dose not reported) and metformin may have additive effects on decreasing the prevalence of MetS [35]. These conflicting results could possibly be explained by the difference in study design and methodology. The lack of a systematic comparison of combined treatments with either exercise or metformin, the deficiency in reporting or control of respective exercise and metformin doses, and the difference in intervention duration between studies, limits our ability to derive a definitive conclusion.

Nevertheless, it should be noted that tracking the temporal change in MetS z-score as a biomarker of cardiometabolic risk, as seen in the present study and that of Malin et al. [15], has been shown to be more strongly associated with the incidence of T2D and CVD compared to other common determinants of CVD risk change over time [36]. This places more emphasis on the clinical significance of our findings and those of Malin et al. [15], suggesting no additional benefit of metformin intake beyond exercise participation to reduce MetS severity and thus development of T2D and CVD. An additional strength of the present study is the specific examination of the inter-individual MetS z-score changes in response to the treatments, which could be argued as a more robust methodology to establish clinical significance relative to simply relying on a pooled average of results. Moreover, when comparing the interaction of metformin and different exercise volumes, the present study indicates that metformin may blunt the positive impact of a high-volume HIIT (4HIIT) on MetS severity amelioration. This can also be deemed as a clinically significant finding considering that 4HIIT has consistently been reported to be a superior treatment in the reduction of cardiometabolic risk factors relative to MICT [37,38,39], specifically in those diagnosed with MetS [40].

### Limitations

The strength of the study was limited by only 18 of the 65 participants taking metformin and only six of these performing high volume HIIT. Another limitation is the lack of standardised dosage and adherence record of metformin taken. Future studies utilising a systematic approach to the prescription of metformin and exercise dosages are therefore warranted. It should also be highlighted that those who reported to be taking metformin had a confirmed diagnosis of T2D, whereas those who were not taking metformin were classified as non-T2D. It would therefore be interesting to investigate if similar results would be evident if the combination of metformin and different exercise volumes were applied to those who have not yet developed T2D. Another limitation of this study was that a proportion of prescribed exercise was conducted unsupervised, relying on self-reported HR and RPE data. However, it could be inferred that the implementation HR monitors, RPE scales and reporting logs may have mitigated errors of self-reporting. Moreover, given the high adherence rate to the exercise intervention in the present study, it could be argued that the inclusion of unsupervised exercise sessions may also be considered a strength of the study, indicating that these types of exercise can be implemented as a self-management tool, with or without medication, to counteract the severity of MetS. It should also be noted that the use of %HRpeak instead of %HRreserve to prescribe and monitor exercise intensity could have limited the replication and applicability of our results. However, we wanted to simplify the methodology of our study for easy translation of our exercise protocols and thus self-management of individuals in an unsupervised environment. It could also be argued that the instruction employed to monitor rate of perceived exertion using the Borg scale during exercise could have interrupted the participants’ focus on acquiring the desired intensity during the exercise session [41], influencing the replication and the applicability of our results. Lastly, given that we undertook the graded exercise tests via two modes (treadmill vs. cycle ergometers), caution should be made when interpreting our CRF results.

## 5. Conclusions

While our sample size is small, our findings suggest that the effect of high volume HIIT on MetS severity may be blunted in individuals with MetS who are taking metformin medication. These findings need to be confirmed in a larger study.

## Figures and Tables

**Table 1:** Participant characteristics of those not taking or taking metformin.

Participant Characteristics/Outcome Variable	Not taking Metformin (*n* = 47)		Taking Metformin (*n* = 18)		
	Baseline	Post	Baseline	Post	Between Group Diff (*p*-Value)
Age (year)	56 ± 9	--	59 ± 7	--	0.13
T2D, %	17	--	100	--	<0.001
Body mass (kg)	96 ± 19	94 ± 19	98 ± 20	97 ± 20	0.61
BMI (kg/m^2^)	32 ± 5	32 ± 5	33 ± 6	32 ± 6	0.59
Waist circumference (cm)	104 ± 12	102 ± 11	108 ± 12	105 ± 12	0.82
Systolic BP (mm Hg)	132 ± 13	124 ± 10	133 ± 13	130 ± 13	0.03
Diastolic BP (mm Hg)	85 ± 9	81 ± 7	82 ± 7	79 ± 8	0.55
Triglycerides (mmol/L)	1.9 ± 1.0	1.8 ± 0.9	2.0 ± 0.7	1.9 ± 1.0	0.88
HDL-C (mmol/L)	1.2 ± 0.4	1.2 ± 0.4	1.0 ± 0.2	1.1 ± 0.3	0.83
Glucose (mmol/L)	5.9 ± 1.2#	5.8 ± 1.3	8.3 ± 2.5	7.9 ± 2.4	0.48
HOMA-IR	2.3 ± 1.1	1.9 ± 1.1	1.9 ± 0.9	1.6 ± 0.8	0.79
MetS z-score	1.6 ± 2.3#	0.8 ± 2.1	4.2 ± 2.5	3.0 ± 3.2	0.81
VO_2_peak (mL/kg/min)	26.4 ± 6.6	28.4 ± 6.9	24.9 ± 6.6	27.4 ± 6.6	0.78

**#** Baseline value significantly different from participants taking Metformin.

**Table 2:** Participants not taking metformin.

Participant Characteristics/Outcome Variable	MICT (*n* = 16)		4HIIT (*n* = 16)		1HIIT (*n* = 15)		
	Baseline	Post	Baseline	Post	Baseline	Post	Between Group Difference (*p*-Value)
Age (year)	56 ± 9	--	55 ± 10	--	57 ± 9	--	0.72
T2D, %	6	--	19	--	27	--	0.31
Body mass (kg)	99 ± 20	98 ± 21	97 ± 16	96 ± 16	91 ± 20	89 ± 19	0.81
BMI (kg/m^2^)	32 ± 6	32 ± 6	33 ± 4	33 ± 4	30 ± 5	30 ± 4	0.87
Waist circumference (cm)	107 ± 13	105 ± 14	104 ± 10	103 ± 8	102 ± 13	99 ± 11	0.43
Systolic BP (mm Hg)	131 ± 15	124 ± 10	131 ± 12	126 ± 11	133 ± 13	123 ± 10	0.26
Diastolic BP (mm Hg)	87 ± 11	82 ± 8	85 ± 7	81 ± 7	83 ± 7	80 ± 4	0.86
Triglycerides (mmol/L)	1.7 ± 0.8	1.7 ± 1.0	2.4 ± 1.3	2.1 ± 1.1	1.7 ± 0.7	1.6 ± 0.6	0.81
HDL-C (mmol/L)	1.2 ± 0.4	1.2 ± 0.3	1.0 ± 0.4	1.2 ± 0.4	1.2 ± 0.5	1.3 ± 0.5	0.39
Glucose (mmol/L)	5.8 ± 0.8	5.5 ± 0.6	6.0 ± 1.9	6.1 ± 1.9	5.8 ± 0.9	5.9 ± 0.7	0.24
HOMA-IR	2.4 ± 1.4	1.8 ± 1.2	2.2 ± 1.0	1.8 ± 0.8	2.2 ± 1.0	2.2 ± 1.3	0.17
MetS z-score	1.5 ± 2.0	0.6 ± 1.9	2.1 ± 2.6	1.3 ± 2.6	1.3 ± 2.3	0.3 ± 1.7	0.60
VO_2_peak (mL/kg/min)	27.9 ± 7.3	28.9 ± 6.7	24.5 ± 4.5	27.9 ± 6.5	26.4 ± 7.5	28.3 ± 7.8	0.39

**Table 3:** Participants taking metformin.

Outcome Variable	MICT (*n* = 4)		4HIIT (*n* = 6)		1HIIT (*n* = 8)		
	Baseline	Post	Baseline	Post	Baseline	Post	Between Group Difference (*p*-Value)
Age (year)	63 ± 6	--	58 ± 9	--	58 ± 4	--	0.44
T2D, %	100	--	100	--	100	--	>0.05
Body mass (kg)	103 ± 11	98 ± 15	102 ± 20	101 ± 20	93 ± 24	92 ± 24	0.46
BMI (kg/m^2^)	33 ± 4	32 ± 4	34 ± 7	34 ± 8	32 ± 7	31 ± 7	0.73
Waist circumference (cm)	111 ± 9	107 ± 9	109 ± 12	107 ± 14	105 ± 13	102 ± 13	0.56
Systolic BP (mm Hg)	136 ± 10	129 ± 15	123 ± 8	129 ± 6	138 ± 14	132 ± 18	0.14
Diastolic BP (mm Hg)	84 ± 5	78 ± 7	82 ± 7	79 ± 7	80 ± 7	79 ± 10	0.94
Triglycerides (mmol/L)	1.8 ± 0.7	1.3 ± 0.4	2.0 ± 0.9	2.3 ± 1.5	2.1 ± 0.7	1.8 ± 0.6	0.17
HDL-C (mmol/L)	1.0 ± 0.4	1.1 ± 0.5	0.9 ± 0.2	0.9 ± 0.02	1.0 ± 0.2	1.2 ± 0.2	0.13
Glucose (mmol/L)	6.4 ± 1.0	6.9 ± 2.3	8.6 ± 3.0	8.5 ± 2.3	9.1 ± 2.4	8.1 ± 2.8	0.64
HOMA-IR	1.7 ± 0.8	1.3 ± 0.7	1.9 ± 0.7	1.9 ± 0.8	1.9 ± 1.1	1.5 ± 0.9	0.12
MetS z-score	2.7 ± 1.3	1.7 ± 2.6	4.3 ± 1.9	4.2 ± 1.9	4.8 ± 3.2	2.8 ± 4.1	0.26
VO_2_peak (mL/kg/min)	25.6 ± 8.6	25.9 ± 6.6	24.7 ± 8.7	28.8 ± 9.6	24.6 ± 4.4	27.1 ± 4.3	0.33
